# Practical Notes on Diagnosis and Treatment

**Published:** 1910-03-05

**Authors:** 


### Practical Notes on Diagnosis and Treatment.

### Tuberculous Rales.

If you examine a patient, say, on admission to hospital, and find crepitations on inspiration and expiration, and if in one month's time you find that the expiratory crepitations have disappeared, you can say emphatically that the process is one of amelioration.—*Dr. S. H. Habershon.*

### Whooping Cough.

Quinine is one of the most valuable drugs in this complaint; the dose may be reckoned as $\frac{1}{6}$ grain for each month of the child's age, and $1\frac{1}{2}$ grains for each year, given three times during the day. More than 6 grains for a dose is not necessary for older children.

### Ulcerative Stomatitis.

This affection is more common in children than is supposed, and is the cause of considerable pain and constitutional disturbance. It is probable that it is an infectious disorder, and any source of infection should be searched for. The treatment consists in calomel and saline, demulcent food, and potass. chlorate with iron well diluted. Local treatment may be confined to a mouth wash of permanganate of potassium.—*Dr. G. A. Sutherland.*

### Gastric Ulcer.

The administration of calcium chloride has been followed by prompt relief of symptoms in some cases of gastric ulcer.—*Dr. G. Parker.*

### Pain on Micturition.

If pain is increased during the act and diminishes afterwards this points to acute cystitis. If the pain is more severe at the end of the act and increased on movement this suggests stone.

### Delayed Chloroform Poisoning.

I consider that the vomiting which occurs after the administration of chloroform to be essentially toxæmic, due to the profound depression of liver function with consequent diminution of its antitoxic function during the administration. This depression is further weakened by the enforced abstention from food, and this may in individual cases be carried too far, and it is, in my opinion, largely responsible for the fatal effects of delayed chloroform-poisoning in exceptional cases. Such effects might be avoided if the patient, instead of being starved, were to have an easily digestible meal, well sweetened, three hours before operation.—*Dr. Wm. Hunter.*

